# Stress-coping strategies among medical residents in Saudi Arabia: A cross-sectional national study

**DOI:** 10.12669/pjms.313.7490

**Published:** 2015

**Authors:** Fahad D. Alosaimi, Auroabah Almufleh, Sana Kazim, Bandar Aladwani

**Affiliations:** 1 Fahad D Alosaimi, MD. Assistant Professor of King Saud University, Riyadh, KSA. Psychiatry & Psychosomatic Medicine Consultant, Psychiatry Dept. # 55, King Khalid University Hospital, Riyadh, Kingdom of Saudi Arabia; 2 Aroba Almufleh, Psychiatry Resident, Department of Psychiatry, Saudi Commission for Health Specialties, Riyadh, Saudi Arabia; 3 Sana Kazim, Psychiatry Resident, Department of Psychiatry, Saudi Commission for Health Specialties, Riyadh, Saudi Arabia; 4 Bandar Aladwani, Psychiatry Resident, Department of Psychiatry, Saudi Commission for Health Specialties, Riyadh, Saudi Arabia

**Keywords:** Residents, Coping, Stress, Brief COPE, Perceived stress scale, Saudi Arabia

## Abstract

**Objectives:**

Maladaptive stress-coping strategies have been linked to reduced quality of life, psychiatric disorders, and reduced work performance among residents or physicians. This study aimed to examine stress-coping strategies among medical residents in Saudi Arabia and their association with stress levels and important personal characteristics.

**Methods:**

This cross-sectional study was conducted between May and October 2012. Residents of different specialties were recruited from a national database. Stress-coping strategies were assessed using the 28-item brief coping scale (BCS), while stress was assessed using the perceived stress scale (PSS).

**Results:**

Nine hundred seventeen residents completed both BCS and PSS assessments. Almost 55% of participants were males, 88% were Saudi, 58% were married, and 15% had positive history of psychiatric disorders. The adaptive stress-coping strategy with the highest score was religion, followed by planning, acceptance, and active coping. The maladaptive stress-coping strategy with the highest score was self-blame, followed by self-distraction, and venting. Maladaptive stress-coping strategies were associated with high stress level, female gender, and history of psychiatric disorders. Stress-coping strategies were not correlated/associated with age, presence of major medical illnesses, or stress management education/training.

**Conclusion:**

Adaptive stress-coping strategies were more frequently used among a sample of residents in Saudi Arabia than maladaptive stress-coping strategies, with higher use of religion in coping than previously reported. To avoid potential negative impact on resident well-being, future studies among residents should aim to identify the type of stress management program that most positively impacts stress-coping skills.

## INTRODUCTION

Medical residents are often working under stressful conditions, such as prolonged working hours and high job demands.^1^ A considerable number of residents in different specialties suffer from high levels of perceived stress and burnout symptoms.^2^,^3^ These may negatively impact the physical and psychological well-being of the residents, as well as the quality of the patient care^4^ Although stress and burnout among residents has been well studied, little work has addressed work-related coping strategies among residents.^5^-^8^

Exposure to stressful working conditions in the absence of adequate stress-coping strategies may lead to burnout or depression.^9^ Additionally, the negative effect of stress on work performance is mediated by the type of stress-coping strategies used and certain personality traits.^10^,^11^ Stress-coping strategies can be grouped into adaptive and maladaptive strategies,^12^-^15^ as well as problem-focused and emotion-focused strategies.^14^ However, the positivity of any stress-coping strategy is dependent on the stressor context and situation.^16^ Maladaptive stress-coping strategies, such as alcohol/drugs use, denial, disengagement, venting and self-blame, have been associated with reduced quality of life^4^, depression and anxiety,^13^ and reduced work performance^6^ among residents and other healthcare providers.

Reflecting the great interest in securing sufficient number of local practicing physicians, the number of recognized residency programs and enrolled residents has recently increased in Saudi Arabia.^17^ However, data examining stress and/or stress-coping strategies among residents in Saudi Arabia are currently severely lacking. We were able to identify only a single study that examined stress and its coping strategies among Saudi dental students.^18^ The objective of the current study was to examine stress-coping strategies among residents in Saudi Arabia and their association with the level of stress and important personal characteristics.

## METHODS

The study population was recruited from a pool of residents registered at the Saudi Commission for Health Specialties (SCHS). Thirty-seven residency and fellowships training programs in multiple health specialties have been recognized by the SCHS. The current study was conducted among medical residents trained in different residency programs in all regions of Saudi Arabia. Interns and fellow were excluded.

This cross-sectional study was conducted between May & October, 2012. It was approved by the institutional review board and the Faculty of Medicine at King Saud University, Riyadh, Saudi Arabia.

A list of all residents registered at SCHS at the time of the study was obtained. Three successive emails were sent to members on this list explaining the study objectives with the study questionnaire and the informed consent form was attached. Out of the 4,000 members on the list, 1,035 returned the email with completed Forms, representing a 25.9% response rate. Subsequently, 57 participants were excluded as they identified themselves as fellows and another 61 participants were excluded because of missing coping and/or perceived stress scales.

### Data collection tool

A self-administrated questionnaire was developed and included socio-demographic characteristics, clinical history, and occupational characteristics. A multi-disciplinary committee covering ethics, psychiatry, and epidemiology validated the questionnaire, which was then piloted on a small number of participants (n = 20). Finally, the wording and suggested answers were modified for some questions based on feedback from the pilot sample. Stress-coping strategies employed in the past month were assessed using the 28-item brief coping scale (BCS).^19^ The scale assesses 14 stress-coping strategies using 28 questions (2 questions per strategy). Each question is answered in a 4-point Likert-type scale. Each question is given a score ranging from 1 to 4 and each strategy is given a score ranging from 2 to 8. Stress-coping strategies were grouped into adaptive or maladaptive strategies, as previously described.^12^ The perception of stress over the past month was assessed using the 10-question perceived stress scale (PSS).^20^,^21^ Each question is answered in a 5-point Likert-type scale and is given a score (ranging from 0 to 4). The PSS score was calculated by summing individual questions’ scores, with higher scores corresponding to higher levels of stress (maximum 40 points). The overall PSS, as well as adaptive and non-adaptive BCS, had good internal consistency among their items, as indicated by overall Cronbach Alpha values of 0.744, 0.837, and 0.684 respectively.

### Statistical Analysis

Data were presented as frequencies and percentages for categorical data and mean and standard deviation (SD) for continuous data. As there is no standard cut-off score to diagnose and/or grade stress,^21^ the score of PSS was categorized into three tertiles: lower tertile, less than 20; middle tertile, between 20 and 24; and upper tertile, more than 24. To examine the associations between categorical variables and the scores of coping strategies, one-way analysis of variance (ANOVA) or student t-test (as appropriate) were used. To examine the correlations between continuous variables and the scores of coping strategies, Spearman’s correlation was used. All P-values were two-tailed. P-value <0.05 was considered as significant. SPSS software (release 20.0, SPSS Inc., Chicago, U.S.) was used for statistical analyses.

## RESULTS

A total of 917 residents completed both coping and perceived stress scales. The mean age of the participating residents was 28.4±3.0 years. They were composed of approximately 55% males, 88% were Saudi, and 58% were married. The majority (68%) had an income of SR 15,000 to 19,999, with 46% satisfied with their income and 36% dissatisfied with their income. Approximately 15% (N=132) had a positive history of psychiatric disorders and 8% (N=73) had major medical illnesses, with an average of 1.3±0.6 diseases. Only 8% (N=77) received education or training in stress management. The responses of the study participants to the 28 items of the BCS are shown in Table-I. Among the adaptive stress-coping items, finding comfort in religion or spiritual beliefs, praying or meditating, thinking hard about the steps to be taken, and accepting the reality were the most frequently employed adaptive stress-coping strategies. Among the maladaptive stress-coping items, self-distraction such as going to movies or watching TV, criticizing oneself, and blaming oneself were the most frequent maladaptive stress-coping strategies.

**Table-I:** Items of brief coping scale among medical residents in Saudi Arabia (N=917).

		Not at all	Little bit	Medium amount	A lot
1	I’ve been turning to work or other activities to take my mind off things	226 (24.7%)	315 (34.5%)	222 (24.3%)	151 (16.5%)
2	I’ve been concentrating my efforts on doing something about the situation I’m in	74 (8.1%)	257 (28.1%)	336 (36.8%)	246 (26.9%)
3	I’ve been saying to myself “this isn’t real”	445 (48.8%)	226 (24.8%)	173 (19.0%)	68 (7.5%)
4	I’ve been using alcohol or other drugs to make myself feel better	808 (88.5%)	44 (4.8%)	31 (3.4%)	30 (3.3%)
5	I’ve been getting emotional support from others	161 (17.6%)	323 (35.2%)	259 (28.2%)	174 (19.0%)
6	I’ve been giving up trying to deal with it	246 (26.9%)	357 (39.1%)	237 (26.0%)	73 (8.0%)
7	I’ve been taking action to try to make the situation better	44 (4.8%)	198 (21.7%)	450 (49.3%)	220 (24.1%)
8	I’ve been refusing to believe that it has happened	457 (50.1%)	282 (30.9%)	119 (13.0%)	55 (6.0%)
9	I’ve been saying things to let my unpleasant feelings escape	200 (21.9%)	316 (34.6%)	266 (29.1%)	131 (14.3%)
10	I’ve been getting help and advice from other people	110 (12.0%)	306 (33.4%)	308 (33.6%)	192 (21.0%)
11	I’ve been using alcohol or other drugs to help me get through it	871 (95.1%)	36 (3.9%)	6 (0.7%)	3 (0.3%)
12	I’ve been trying to see it in a different light, to make it seem more positive	98 (10.7%)	264 (28.9%)	370 (40.4%)	183 (20.0%)
13	I’ve been criticizing myself	66 (7.3%)	267 (29.5%)	330 (36.5%)	241 (26.7%)
14	I’ve been trying to come up with a strategy about what to do	42 (4.6%)	253 (27.7%)	390 (42.8%)	227 (24.9%)
15	I’ve been getting comfort and understanding from someone	114 (12.5%)	313 (34.4%)	347 (38.1%)	136 (14.9%)
16	I’ve been giving up the attempt to cope	260 (28.8%)	362 (40.1%)	235 (26.0%)	46 (5.1%)
17	I’ve been looking for something good in what is happening	59 (6.5%)	274 (30.0%)	384 (42.1%)	195 (21.4%)
18	I’ve been making jokes about it	204 (22.4%)	289 (31.8%)	273 (30.0%)	143 (15.7%)
19	I’ve been doing something to think about it less, such as going to movies, watching TV, reading, daydreaming, sleeping, or shopping	95 (10.4%)	193 (21.1%)	290 (31.8%)	335 (36.7%)
20	I’ve been accepting the reality of the fact that it has happened	55 (6.0%)	213 (23.3%)	384 (42.0%)	262 (28.7%)
21	I’ve been expressing my negative feelings	73 (8.0%)	349 (38.4%)	343 (37.7%)	145 (15.9%)
22	I’ve been trying to find comfort in my religion or spiritual beliefs	57 (6.2%)	182 (19.9%)	333 (36.4%)	342 (37.4%)
23	I’ve been trying to get advice or help from other people about what to do	77 (8.4%)	295 (32.2%)	350 (38.3%)	193 (21.1%)
24	I’ve been learning to live with it	43 (4.7%)	250 (27.4%)	441 (48.4%)	178 (19.5%)
25	I’ve been thinking hard about what steps to take	33 (3.6%)	211 (23.2%)	387 (42.6%)	278 (30.6%)
26	I’ve been blaming myself for things that happened	127 (13.9%)	315 (34.5%)	244 (26.7%)	228 (24.9%)
27	I’ve been praying or meditating	61 (6.7%)	217 (23.8%)	334 (36.6%)	300 (32.9%)
28	I’ve been making fun of the situation	223 (24.5%)	323 (35.4%)	260 (28.5%)	106 (11.6%)

The scores of adaptive and maladaptive stress-coping strategies are shown in Table-II. The adaptive stress-coping strategy with the highest score was religion followed by planning, acceptance and active coping. The maladaptive stress-coping strategy with the highest score was self-blame, followed by self-distraction venting and behavioral disengagement. Out of a maximum possible score of 40, the average PSS was 22.0±5.1.

**Table-II:** Stress-coping strategies and perceived stress scale among medical residents in Saudi Arabia (N=917).

	Maximum possible points	Absolute score		Relative score*	
		Mean	SD	Mean	SD
Adaptive stress-coping strategies	64	43.7	7.9	68.3%	12.3%
Active coping (2 and 7)	8	5.7	1.5	71.6%	18.9%
Instrumental support (10 and 23)	8	5.4	1.6	66.8%	20.1%
Planning (14 and 25)	8	5.8	1.4	73.1%	17.6%
Acceptance (20 and 24)	8	5.8	1.4	71.8%	17.6%
Emotional support (5 and 15)	8	5.0	1.7	62.8%	20.7%
Humor (18 and 28)	8	4.6	1.8	58.0%	23.0%
Positive reframing (12 and 17)	8	5.5	1.5	68.3%	18.7%
Religion (22 and 27)	8	6.0	1.6	74.9%	20.2%
Maladaptive stress-coping strategies	48	25.6	5.1	53.4%	10.7%
Behavioral disengagement (6 and 16)	8	4.2	1.5	52.3%	19.0%
Denial (3 and 8)	8	3.6	1.6	44.8%	19.7%
Self-distraction (1 and 19)	8	5.3	1.6	65.7%	19.6%
Self-blame (13 and 26)	8	5.4	1.7	67.5%	20.9%
Substance use (4 and 11)	8	2.3	0.8	28.4%	9.9%
Venting (9 and 21)	8	4.9	1.4	61.8%	18.0%
Perceived stress scale	40	22.0	5.1	55.1%	12.7%

* Out of maximum possible score.

The associations and correlations between perceived stress levels and stress-coping strategies are shown in Table-III. Residents with the lowest stress level (lower tertile of PSS) had the highest adaptive stress-coping scores. This was true for positive reframing, humor, planning, and overall adaptive stress-coping strategies. Additionally, PSS had significant negative correlations with the scores of positive reframing, humor, acceptance, active coping, and overall adaptive stress-coping strategies. Whereas residents with the highest stress level (upper tertile of PSS) had the highest maladaptive stress-coping scores and PSS had significant positive correlations with maladaptive stress-coping scores. With the exception of substance use, these associations and correlations were significant for all individual and overall maladaptive stress-coping strategies (p<0.001 for each).

**Table-III:** Association and correlation between perceived stress levels and stress-coping strategies among medical residents in Saudi Arabia (N=917).

Perceived stress scale						
	Tertile values (mean ±SD)				Correlation	
	Low (<20)	Medium (20-24)	High (>24)	p-value	Spearman’s rho	p-value
Adaptive stress-coping strategies	44.82±7.49	43.29±8.06	43.21±7.95	0.021	-0.10	0.003
Active coping (2 & 7)	5.88±1.55	5.68±1.41	5.62±1.57	0.092	-0.09	0.008
Instrumental support (10 & 23)	5.34±1.53	5.34±1.55	5.37±1.75	0.969	0.01	0.724
Planning (14 & 25)	5.95±1.33	5.69±1.48	5.92±1.40	0.044	0.01	0.820
Acceptance (20 & 24)	5.90±1.37	5.72±1.42	5.63±1.42	0.061	-0.12	<0.001
Emotional support (5 & 15)	5.14±1.64	4.97±1.60	4.96±1.73	0.323	-0.04	0.206
Humor (18 & 28)	4.95±1.73	4.59±1.74	4.41±2.01	0.002	-0.14	<0.001
Positive reframing (12 & 17)	5.76±1.45	5.40±1.47	5.24±1.53	<0.001	-0.13	<0.001
Religion (22 & 27)	5.95±1.70	5.97±1.53	6.06±1.62	0.663	0.03	0.366
Maladaptive stress-coping strategies	23.15±4.47	25.55±5.16	28.10±4.44	<0.001	0.42	<0.001
Behavioral disengagement (6 & 16)	3.67±1.47	4.11±1.48	4.75±1.42	<0.001	0.31	<0.001
Denial (3 & 8)	3.13±1.29	3.58±1.60	4.01±1.67	<0.001	0.23	<0.001
Self-distraction (1 & 19)	4.83±1.50	5.28±1.52	5.63±1.59	<0.001	0.21	<0.001
Self-blame (13 & 26)	4.82±1.53	5.33±1.61	6.04±1.66	<0.001	0.32	<0.001
Substance use (4 & 11)	2.23±0.72	2.35±0.88	2.22±0.76	0.094	-0.03	0.446
Venting (9 & 21)	4.46±1.32	4.91±1.43	5.44±1.41	<0.001	0.30	<0.001

The associations and correlations between important characteristics and stress-coping strategies are shown in Table-IV. With the exception of substance use, females had significantly higher individual and overall adaptive and maladaptive stress-coping strategies compared with males. Receiving education or training in stress management was significantly associated with higher religion-coping but not any other stress-coping strategy. Positive history of psychiatric disorders was significantly associated with higher individual and overall maladaptive stress-coping strategies but not associated with any adaptive stress-coping strategies. Although weak, age was negatively correlated with emotional support and self-distraction but positively correlated with behavioral disengagement. The number of major medical illnesses did not have significant correlations with any stress-coping strategy.

**Table-IV:** Association and correlation between important characteristics and stress-coping strategies among medical residents in Saudi Arabia (N=917).

	Score difference		Score difference		Score difference		Score correlation	
	Gender		Education or training in stress management		History of psychiatric disorders		Age	Number of major medical illnesses
	Male	Female	No	Yes	No	Yes	rho	rho*
Adaptive stress-coping strategies	43.00±7.81	44.61±7.90*	43.75±7.84	43.75±8.59	43.83±7.95	43.80±7.84	-0.04	0.03
Active coping (2 & 7)	5.71±1.50	5.74±1.52	5.73±1.52	5.71±1.40	5.77±1.52	5.53±1.57	0.00	0.03
Instrumental support (10 & 23)	5.17±1.57	5.56±1.64*	5.36±1.61	5.25±1.63	5.32±1.61	5.60±1.64	-0.01	-0.02
Planning (14 & 25)	5.82±1.38	5.87±1.44	5.85±1.41	5.82±1.41	5.87±1.43	5.78±1.40	-0.04	0.03
Acceptance (20 & 24)	5.70±1.36	5.80±1.47	5.75±1.42	5.76±1.32	5.75±1.39	5.73±1.52	-0.01	0.03
Emotional support (5 & 15)	4.82±1.59	5.26±1.71*	5.02±1.65	5.04±1.78	5.05±1.65	5.08±1.69	-0.07*	-0.01
Humor (18 & 28)	4.69±1.79	4.57±1.90	4.66±1.86	4.49±1.66	4.66±1.86	4.56±1.70	-0.05	0.03
Positive reframing (12 & 17)	5.35±1.48	5.61±1.51*	5.46±1.49	5.51±1.61	5.48±1.48	5.46±1.59	-0.06	0.01
Religion (22 & 27)	5.82±1.64	6.21±1.56*	5.96±1.61	6.40±1.66*	6.00±1.57	6.06±1.74	0.02	0.06
Maladaptive stress-coping strategies	24.97±5.00	26.45±5.16*	25.68±5.11	25.41±5.24	25.28±4.94	27.77±5.47*	-0.01	0.02
Behavioral disengagement (6 & 16)	4.00±1.46	4.42±1.57*	4.18±1.52	4.23±1.59	4.10±1.49	4.56±1.69*	0.08*	-0.01
Denial (3 & 8)	3.55±1.51	3.63±1.66	3.58±1.58	3.63±1.55	3.50±1.50	4.02±1.82*	-0.02	0.01
Self-distraction (1 & 19)	5.10±1.54	5.44±1.60*	5.27±1.59	5.19±1.40	5.17±1.58	5.65±1.48*	-0.08*	0.04
Self-blame (13 & 26)	5.20±1.62	5.65±1.70*	5.42±1.67	5.32±1.77	5.37±1.69	5.69±1.67*	0.01	0.02
Substance use (4 & 11)	2.35±0.87	2.18±0.69*	2.27±0.80	2.26±0.71	2.22±0.70	2.56±1.17*	0.04	-0.04
Venting (9 & 21)	4.78±1.43	5.14±1.44*	4.96±1.44	4.78±1.47	4.91±1.46	5.29±1.38*	-0.01	0.06

* Significant mean difference using t-test or correlation using Spearman correlation coefficient (rho)

## DISCUSSION

This study examined, for the first time in Saudi Arabia, the stress-coping strategies among residents and their association with the level of stress and important personal characteristics. Data on stress-coping strategies among local residents was absent and this subject has been poorly studied internationally,^5^-^8^ with few studies employing the same coping tool used in the current study.^5^ Similar to previously reported among physicians in the US and Australasia, adaptive stress-coping strategies, which are mostly problem-focused coping, were more frequently used in our residents than maladaptive stress-coping strategies, which are mostly emotion-focused coping.^13^,^14^ This generally healthy coping can be regarded as body resistance to burnout and has also been recorded in students and patients.^12^,^15^ Unlike the limited role of religion as a coping strategy among residents and physicians in the US,^8^,^14^ religion was the most frequently used adaptive stress-coping strategy in the current study. Similarly, religion was a frequently used stress-coping strategy among dental students in Saudi Arabia.^18^ This may reflect the critical role of religion in all aspects of behavior in a conservative country such as Saudi Arabia. Similar to several reports among physicians, planning, acceptance, and active coping in the current study were frequently used adaptive stress-coping strategies.^14^,^22^ While using alcohol/drugs was an infrequently used stress-coping strategy among residents and physicians in the current study.^8^,^14^,^22^

The majority of maladaptive stress-coping strategies in the current study were associated with high stress level, whereas only some adaptive stress-coping were weakly associated with low stress level. The current findings may indicate that exposure to residency stress while using inappropriate adaptive stress-coping strategies can lead to higher perceived stress levels. Almost the same findings were shown in a local study among dental students using the same tools for measuring coping strategies and stress.^18^ Additionally, behavioral disengagement, venting, and denial but not substance use were associated with stress measured using the General Health Questionnaire (GHQ) among 312 Pakistani residents of different specialties.^5^ Furthermore, emotion-focused coping strategies, which are mainly composed of maladaptive stress-coping strategies, were associated with increased psychological stress measured using the GHQ among 489 gastroenterology fellows and attending physicians in the US.^14^

Females in the current study were using stress-coping strategies, especially maladaptive ones, more than males, which might explain the higher stress levels in females than males in the current study (data not shown). The higher tendency of females in different populations to use maladaptive or emotion-focused stress-coping strategies^14^,^18^ and to have more stress^23^ have been previously reported. Similar to the current findings, maladaptive stress-coping strategies in physicians have been linked to the presence of psychiatric diseases such as depression and anxiety,^13^ which can be regarded as failure to adequately resolve stress caused by work.^9^ Unlike previous findings that suggest a positive impact of stress management education on stress-coping,^8^,^24^,^25^ here we identified no association could be detected between stress-coping strategies and receiving education or training in stress management. This may be related to the small number (only 8%) or inadequate content or duration of such education or training.

The current study had many advantages; examining for the first time stress-coping among residents in Saudi Arabia, among a relatively big number of residents across several specialties, recruited from a national database, and using a well-validated stress-coping tool. Nevertheless, we acknowledge a number of limitations. First, the study cross-sectional design precluded the detection of any causal association between stress-coping strategies and perceiving high stress level. Second, being a self-reported study, the possibility of reporting bias, especially for culturally unacceptable coping strategies, cannot be excluded. Finally, the response rate of 26% may affect the generalizability. However, our response rate was comparable to similar studies among residents that used email as a recruitment tool.^26^,^27^

In conclusion, adaptive stress-coping strategies were more frequently used among a sample of residents in Saudi Arabia than maladaptive stress-coping strategies. Religion, planning, acceptance, and active-coping were common adaptive stress-coping strategies, while self-blame, self-distraction, and venting were common maladaptive stress-coping strategies. Maladaptive stress-coping strategies were associated with high stress level, female gender, and history of psychiatric disorders. Future studies among residents may be needed to identify the type of stress management program that most positively impacts stress-coping skills.
